# Supplementary material for: Engineering AvidCARs for combinatorial antigen recognition and reversible control of CAR function
Source: Nat Commun. 2020 Aug 20;11:4166. doi: 10.1038/s41467-020-17970-3 (PMC7441178; doi:10.1038/s41467-020-17970-3)
Supplement: Supplementary file 1 — Supplementary Information [file 41467_2020_17970_MOESM1_ESM.pdf]

Supplementary Information

Engineering AvidCARs for combinatorial antigen recognition and reversible control of CAR function

Salzer et al.

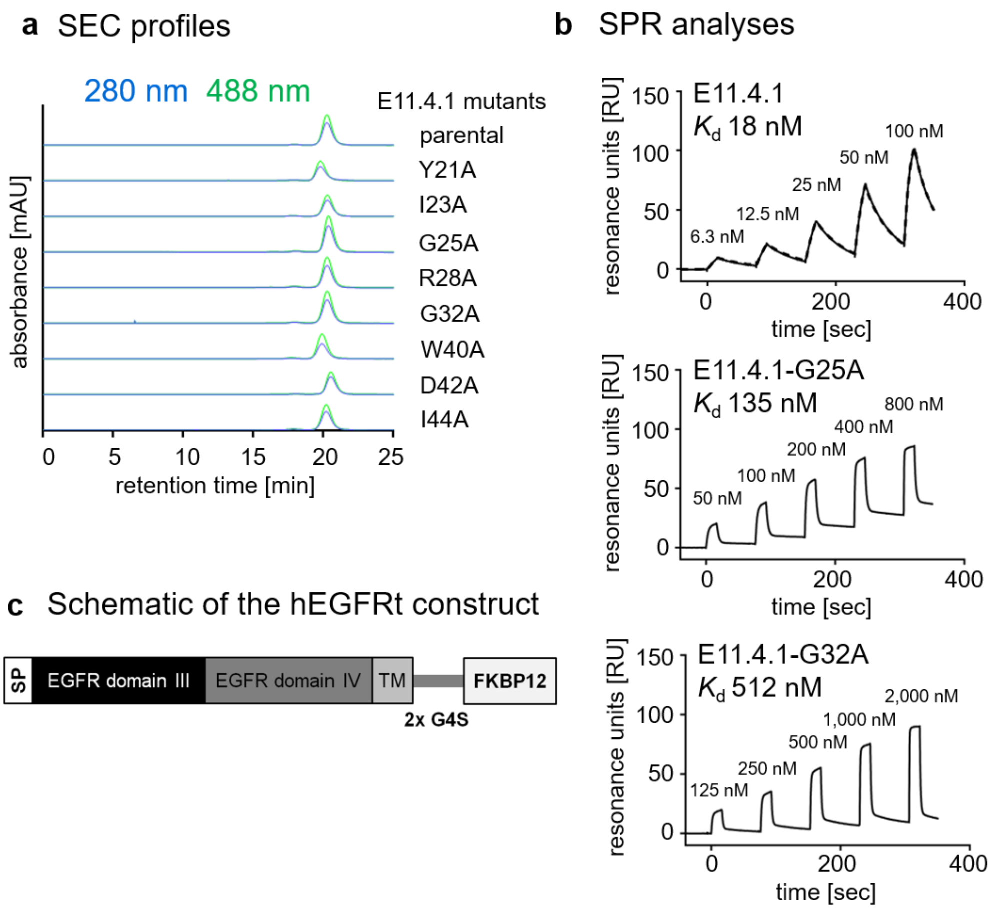

Supplementary Figure 1: Biochemical analysis of hEGFR-specific binder proteins and schematic of the target antigen hEGFRt. (a) Size exclusion chromatography (SEC) profiles of hEGFR-specific binder proteins (E11.4.1 and mutants thereof fused to sfGFP). One representative measurement of 2 independent experiments is shown. **(b)** Single-cycle kinetics SPR experiment with recombinant hEGFR-Fc immobilized on a sensor chip, followed by titration of selected hEGFR-specific binders. K_d values were calculated by steady-state analysis or in case of E11.4.1 by fitting the sensorgram to a 1:1 Langmuir model (dotted line = 1:1 Langmuir fit). Representative diagrams and K_d values of at least 2 independent experiments are shown. **(c)** Schematic of hEGFRt which was used as target antigen in functional assays. The signal peptide (SP) of the granulocyte-macrophage colony-stimulating factor receptor α (GM-CSF-R-α) was fused to a fragment of hEGFR (aa 334 – 675, Uniprot P00533) which was further fused via a GGGGSGGGGS-linker (2 x G4S) to the homodimerization domain FKBP12 (SEQ-ID 20, see Supplementary Data 1).

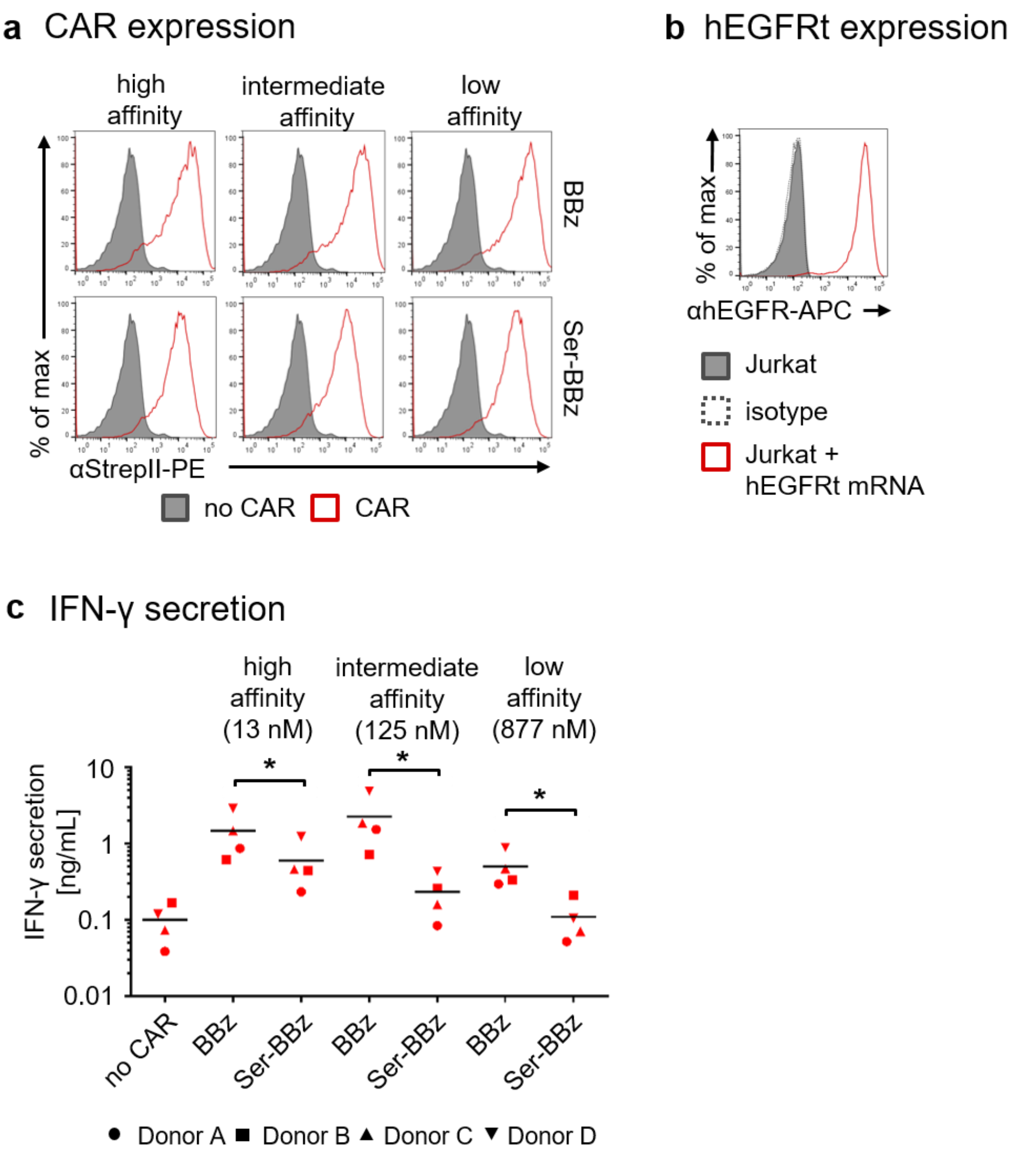

Supplementary Figure 2: Generation of AvidCARs. (a) Expression of the CAR constructs containing hEGFR-specific binders with different affinity. Selected hEGFR-specific binders (high affinity = E11.4.1, intermediate affinity = E11.4.1-G25A, low affinity = E11.4.1-G32A) were fused to the BBz- and the Ser-BBz-CAR backbone. CAR expression in primary human T cells was analyzed by flow cytometry via the Strep II tag present in the CAR constructs [filled gray histogram = mock-T cells (no CAR)]. One representative experiment of 4 independent experiments is shown. **(b)** Expression of the target antigen hEGFRt in Jurkat cells which were used as target cells in the functional assays. Jurkat cells were electroporated with hEGFRt-mRNA and analyzed for expression of hEGFRt by flow cytometry. **(c)** Dependence of CAR function on binder affinity and CAR dimerization. The figure shows the release of

IFN-γ by mock-T cells (no CAR) and T cells expressing the monovalent (Ser-BBz) and bivalent (BBz) CARs (shown in Fig. 2b) with different affinities against hEGFRt, all of which were co-cultured with hEGFRt^{pos} Jurkat cells. (4 independent experiments with 4 different donors; * = $p<0.05$, calculated by two-tailed ratio-paired *t* test). Exact p values are given in Supplementary Data 2. Source data are provided as a Source Data file.

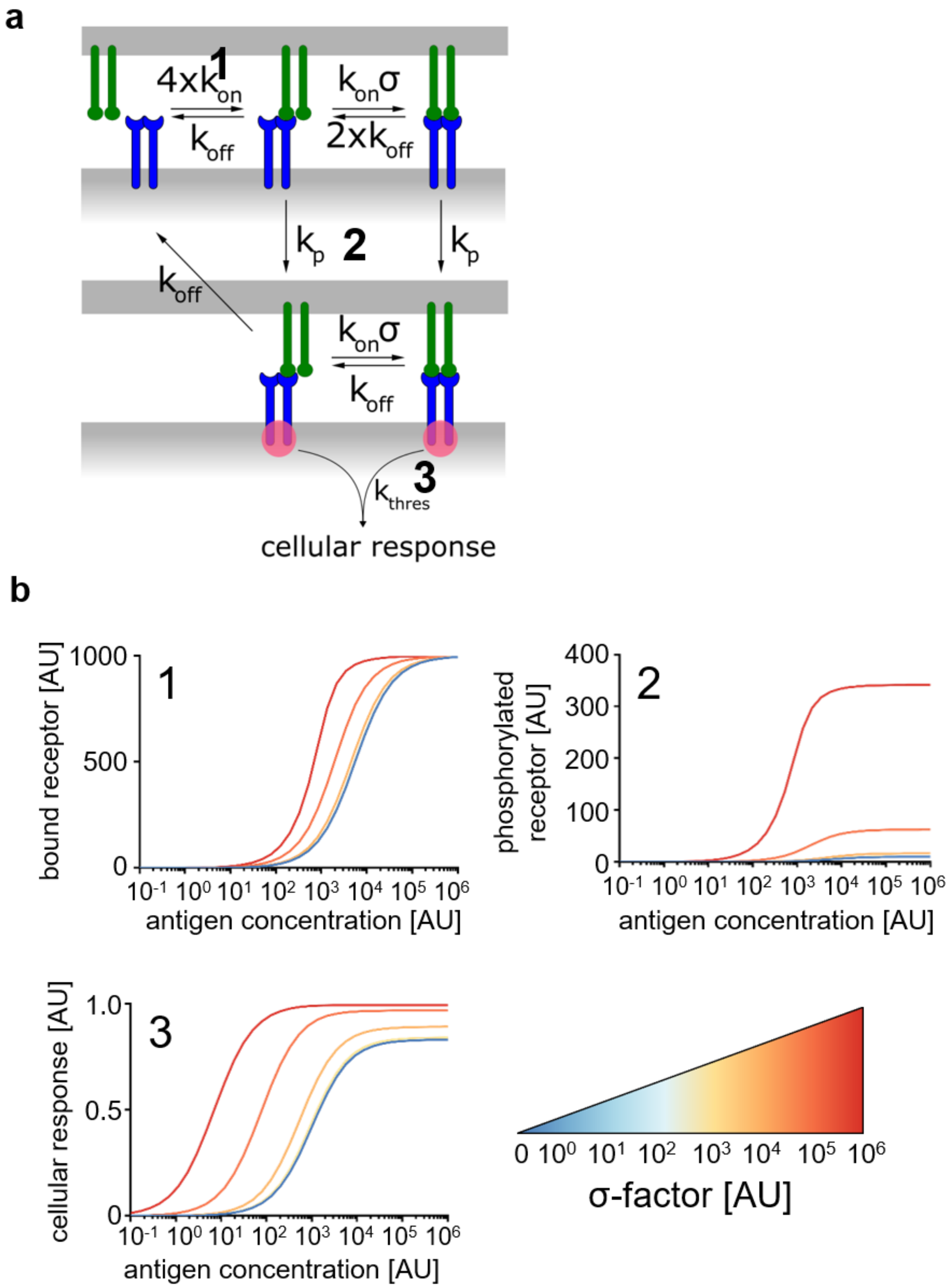

Supplementary Figure 3: Mathematical model of an avidity-controlled CAR. (a) Schematic of the mathematical model describing the interaction between a bivalent antigen (green) and a membrane-bound bivalent CAR (blue) that, upon antigen binding, can undergo kinetic proofreading (e.g. phosphorylation). The initial binding of the CAR to the antigen is described with the usual mass action

binding rates (as indicated) forming a single interaction. The avidity-effect is incorporated by allowing for bivalent binding with this second binding event being modulated by a factor σ. When $\sigma = 0$, the interaction is effectively monovalent (i.e. no avidity enhancement) whereas increasing values of σ exhibit increased avidity. Whenever the CAR is bound to at least one antigen, we allow the complexes to become phosphorylated with the indicated kinetic proofreading rate (k_p), forming a phosphorylated active receptor (red). When the number of active receptors is above a defined threshold, it is assumed that the cell becomes activated. **(b)** Model calculations showing the effect of variation of the σ-factor on the sensitivity of the cells towards the antigen. This is shown as bound total receptor (1), total phosphorylated receptor (2) and cellular response (3).

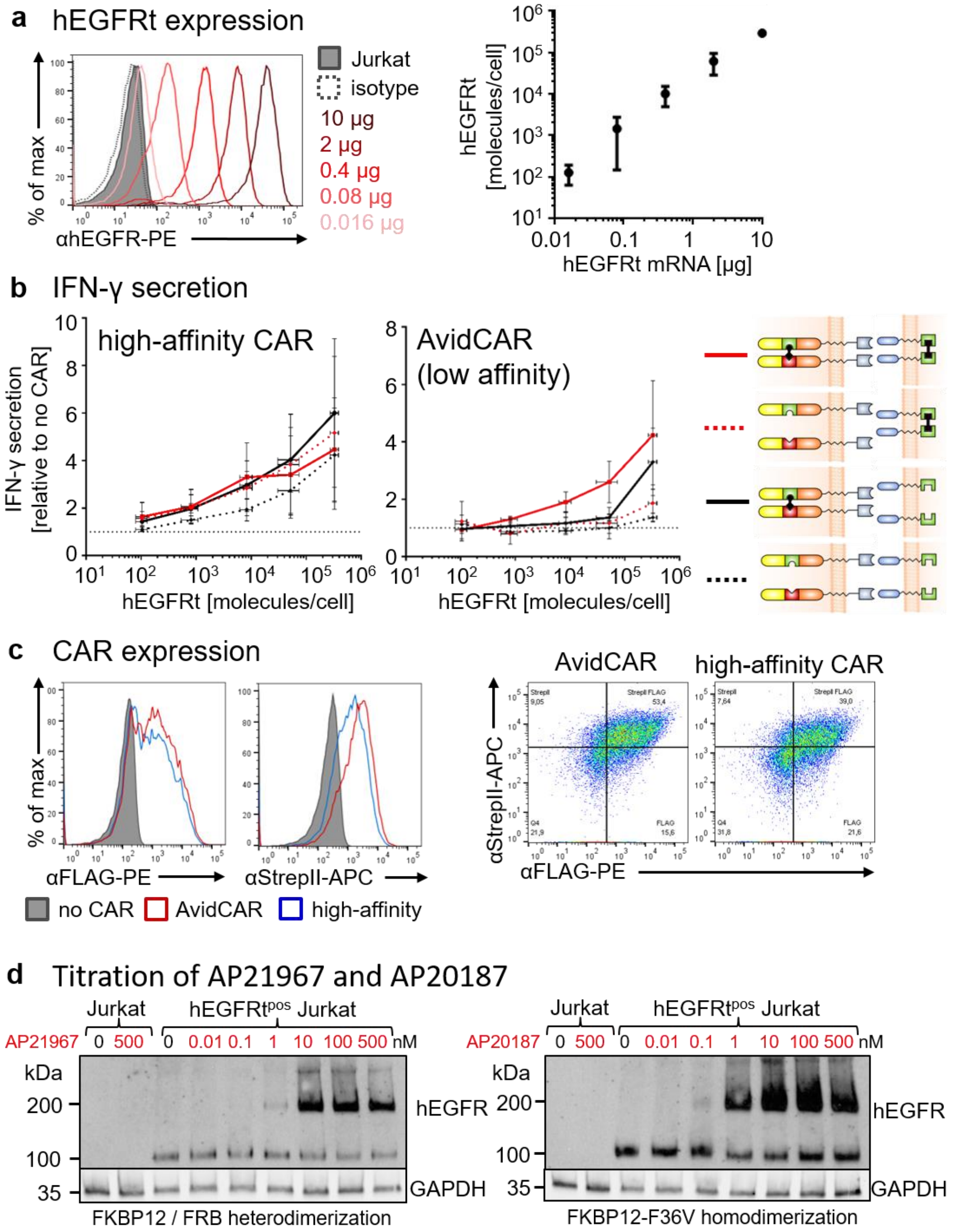

Supplementary Figure 4: Experimental setup for investigating the influence of binder affinity, antigen density, antigen dimerization and CAR dimerization on CAR function. (a) Generation of target cells with different hEGFRt expression levels. Jurkat cells were electroporated with different amounts of hEGFRt-mRNA (as indicated; SEQ-ID 21) and hEGFRt expression was determined by flow cytometry. The histogram overlay on the left illustrates hEGFRt expression in target cells of a representative experiment and the diagram on the right gives the number of hEGFRt molecules per cell (mean ± SD of 3 independent experiments). **(b)** Influence of CAR- and antigen dimerization on IFN-γ release by T

cells expressing either AvidCARs or their high-affinity versions. IFN-γ secretion is depicted for the co-culture experiment shown in Fig. 2e. IFN-γ levels were normalized to the IFN-γ levels obtained with mock T cells (2 independent experiments with 3 different donors). Data are presented as mean values ± SD. **(c)** Expression of each of the two heterodimerizable chains (detected by anti-FLAG and anti-Strep II, respectively) of the high-affinity hEGFR-specific CAR and the respective AvidCAR in primary human T cells. The histogram overlays and the dot plots show the flow cytometric analysis of a representative experiment. **(d)** Determination of the required concentrations of AP21967 for heterodimerization of the domains FKBP12 and FRB, and AP20187 for homodimerization of the domain FKBP12-F36V. The dimerizers were added in different concentrations to Jurkat cells ± expression of hEGFRt fused to the respective domains. The figure shows the Western blot analysis of a representative experiment (after crosslinking with DSS; n=2). Source data are provided as a Source Data file.

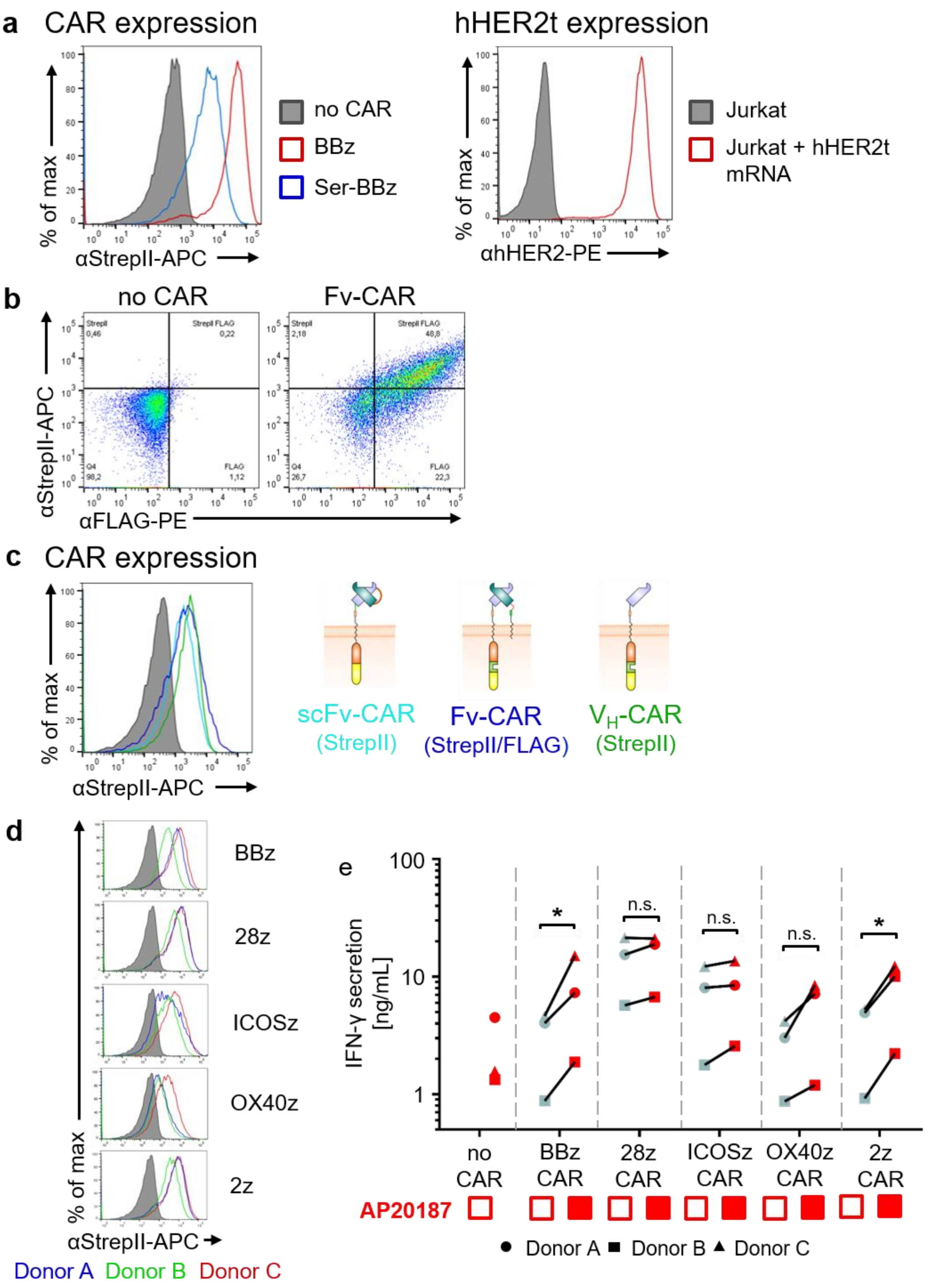

Supplementary Figure 5: ScFv-based low-affinity CARs. (a) Expression of the hHER2-specific scFv-based low-affinity CARs with BBz- or Ser-BBz-backbone (schematically shown in Fig. 3a) in primary human T cells (left) and the target antigen hHER2t in Jurkat cells (right; SEQ-ID 22). The histogram overlays show the flow cytometric analysis of one representative experiment. **(b)** Expression of the

VH- and VL-CAR constructs (together forming a hHER2-specific Fv-based CAR) in primary human T cells. The dot plots show one representative experiment. **(c)** Expression of VH- and or VL-CAR constructs of the Fv-CAR in primary human T cells after adjustment of the mRNA levels for roughly equal expression in the functional analyses shown in Fig. 3e. The histogram overlay shows a representative experiment (filled gray histogram = mock-T cells). **(d and e)** Influence of the co-stimulatory domain on the function of low-affinity CARs. **(d)** Expression of the low-affinity CARs with the different co-stimulatory domains in primary human T cells used in the functional analyses (filled gray histogram = mock-T cells). **(e)** IFN-γ secretion of the co-culture experiment shown in Fig. 3g (2 independent experiments with 3 different donors; * = $p<0.05$, calculated by two-tailed ratio-paired *t* test). Exact p values are given in Supplementary Data 2. Source data are provided as a Source Data file.

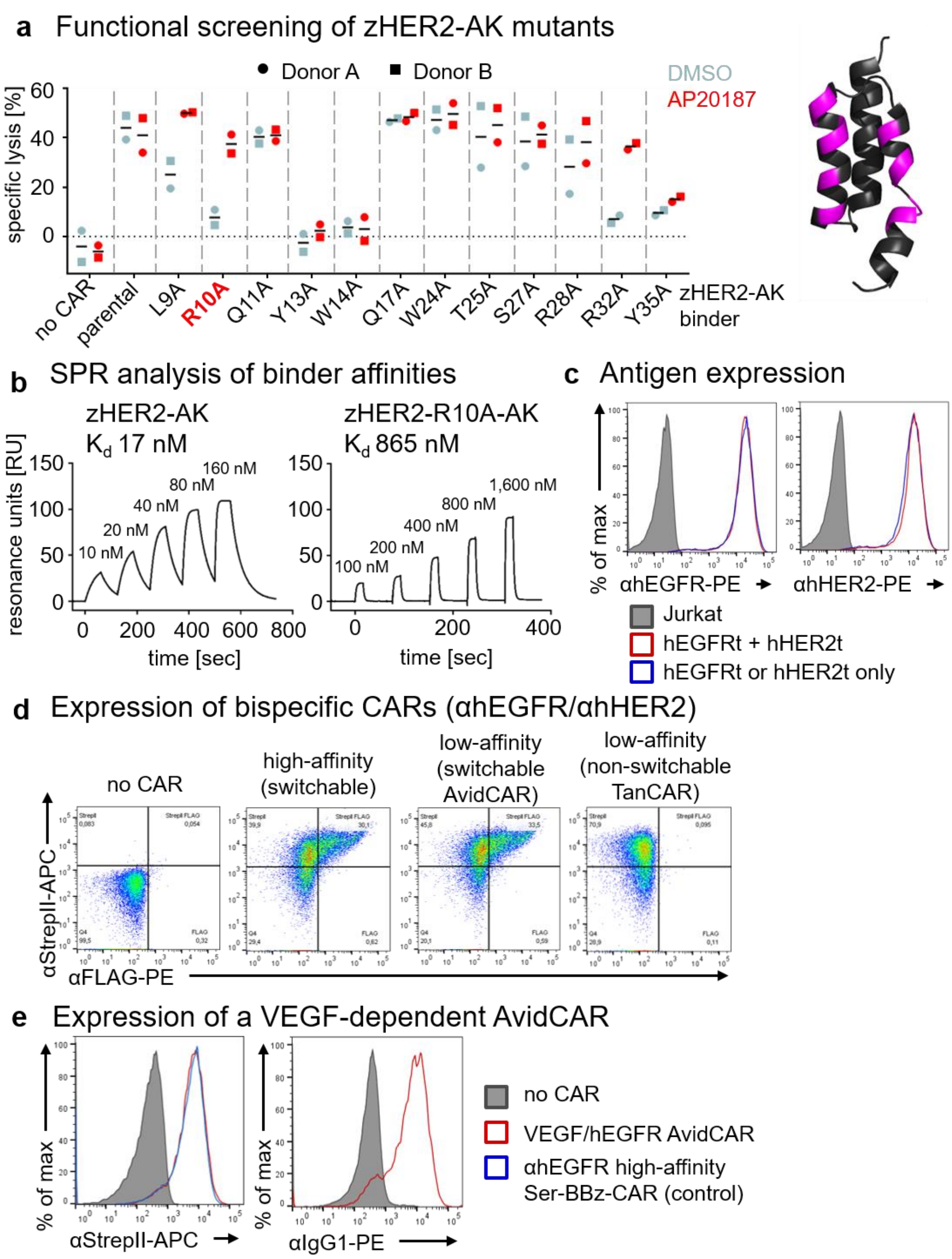

Supplementary Figure 6: AvidCARs with AND-gate function. (a) Functional screening of zHER2-AK-mutants. Primary human T cells either expressing no CAR or CARs with zHER2-AK-mutants (fused to the Ser-BBz backbone with integrated FKBP12-F36V domain) were co-cultured with hHER2t^{pos} Jurkat cells. The figure shows the lytic activity of the T cells after 4 hours of co-culture in presence or absence of the homodimerizer AP20187 as indicated (left). CARs containing the mutants R10A and R32A show strong dependence on the presence of AP20187 (2 independent experiments with 2 different donors). The structure of an affibody scaffold with the mutated amino acid positions (pink) is shown on the

right (PDB 2KZI). **(b)** Single-cycle kinetics SPR experiment with the parental high-affinity zHER2-AK affibody and the selected R10A mutant thereof. Recombinant HER2-Fc was immobilized on a sensor chip, followed by titration with the affibody mutants. K_d values were calculated by steady-state analysis. Representative diagrams and K_d values of 4 independent experiments are shown. **(c)** Target antigen expression in Jurkat cells. Single- and double antigen-positive Jurkat cells were generated by electroporation with hEGFRt and/or hHER2t mRNA. The histograms show one representative experiment. **(d)** Expression of different bispecific CARs (schematically shown in Fig. 4a). The dot plots show one representative experiment. **(e)** Expression of a VEGF-dependent AvidCAR (schematically shown in Fig. 4c). A CAR containing the hEGFR-specific high-affinity rcSso7d-based binder fused to the Ser-BBz backbone was included as a control. Source data are provided as a Source Data file.

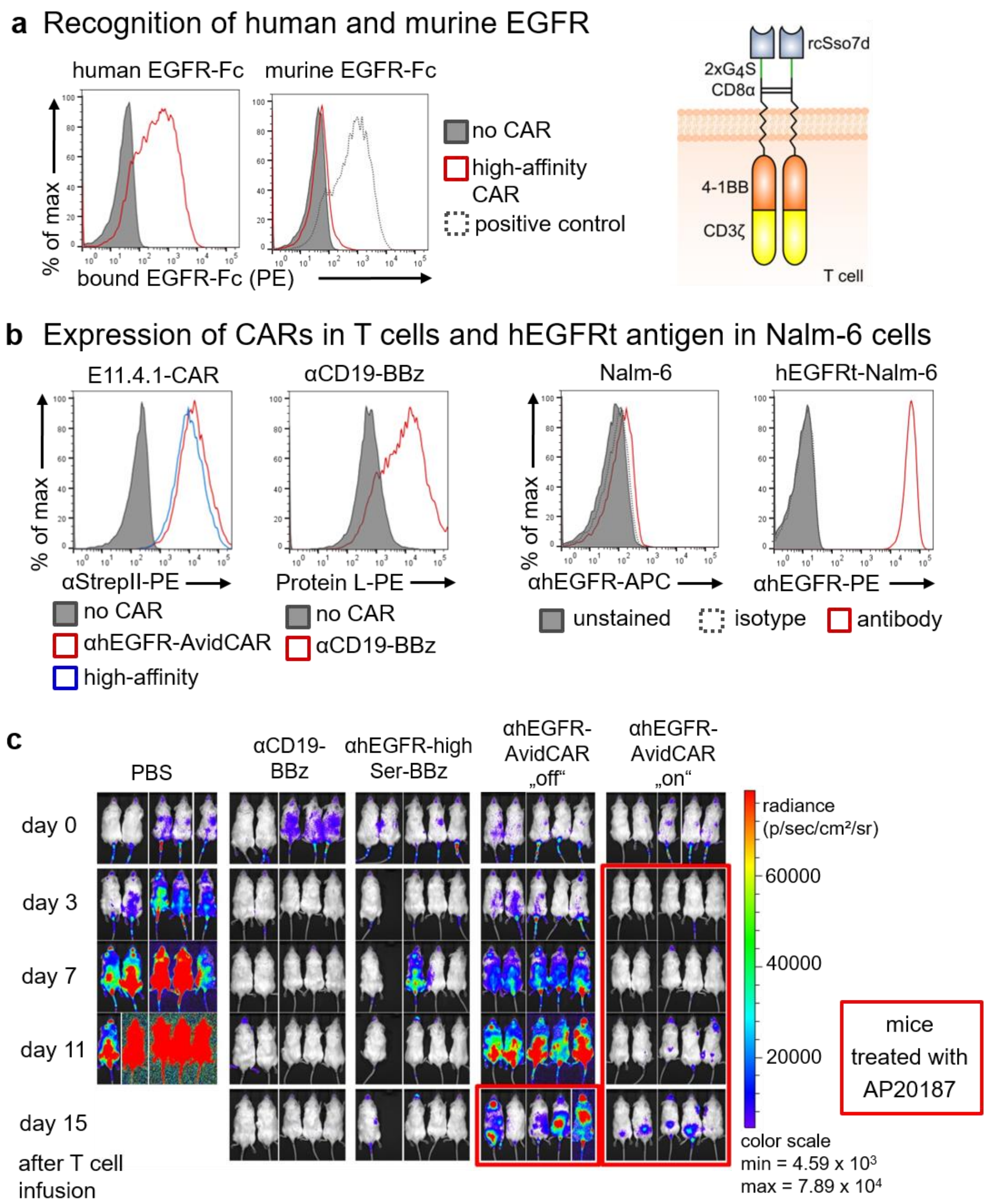

Supplementary Figure 7: Characterization of the hEGFR-specific ON-switch AvidCAR *in vitro* and *in vivo*. **(a)** The selected binder E11.4.1 specifically recognizes human but not murine EGFR. Primary human T cells expressing the high-affinity variant of the rcSso7d-based hEGFR-specific CAR (based on E11.4.1; schematic on the right; SEQ-ID 24) were incubated with recombinant hEGFR-Fc and mEGFR-Fc (histogram overlays; dotted line = positive control for mEGFR-Fc binding). The histograms show one

representative experiment. **(b)** Expression of the lentivirally transduced CAR and antigen constructs in primary human T cells and Nalm-6 target cells, respectively. **(c)** Bioluminescence signal for each mouse of the *in vivo* experiment shown in Fig. 5c.

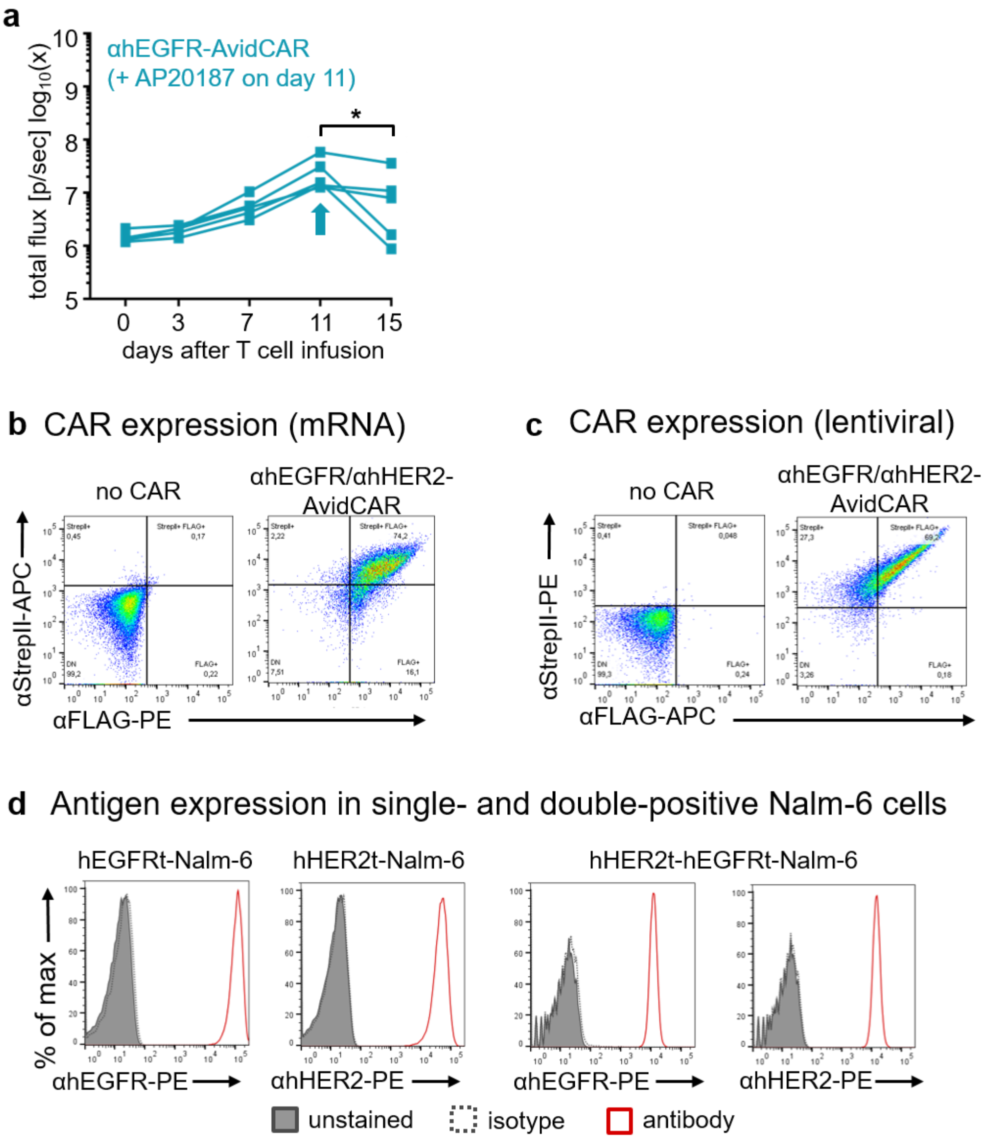

Supplementary Figure 8: *In vivo* characterization of the ON-switch AvidCAR and *in vitro* characterization of the AND-gate AvidCAR. (a) Additional representation of the group of mice with the αhEGFR-AvidCAR-off from Fig. 5c. For better interpretation of the function of the ON-switch AvidCAR, the bioluminescence signal of each mouse of the group with the αhEGFR-AvidCAR-off is shown here individually. The arrow indicates the single injection of 2 mg/kg AP20187. (* = $p<0.05$, calculated by two-tailed paired *t* test). **(b)** Expression of the AND-gate AvidCAR (schematically shown in Fig. 6a) in transiently transfected primary human T cells used for the *in vitro* experiments. **(c)** Expression of the AND-gate AvidCAR in lentivirally transduced primary human T cells used for the *in vivo* experiment. **(d)** Expression of hEGFRt and hHER2t in Nalm-6 cells used for the *in vivo* experiment. Exact p values are given in Supplementary Data 2.

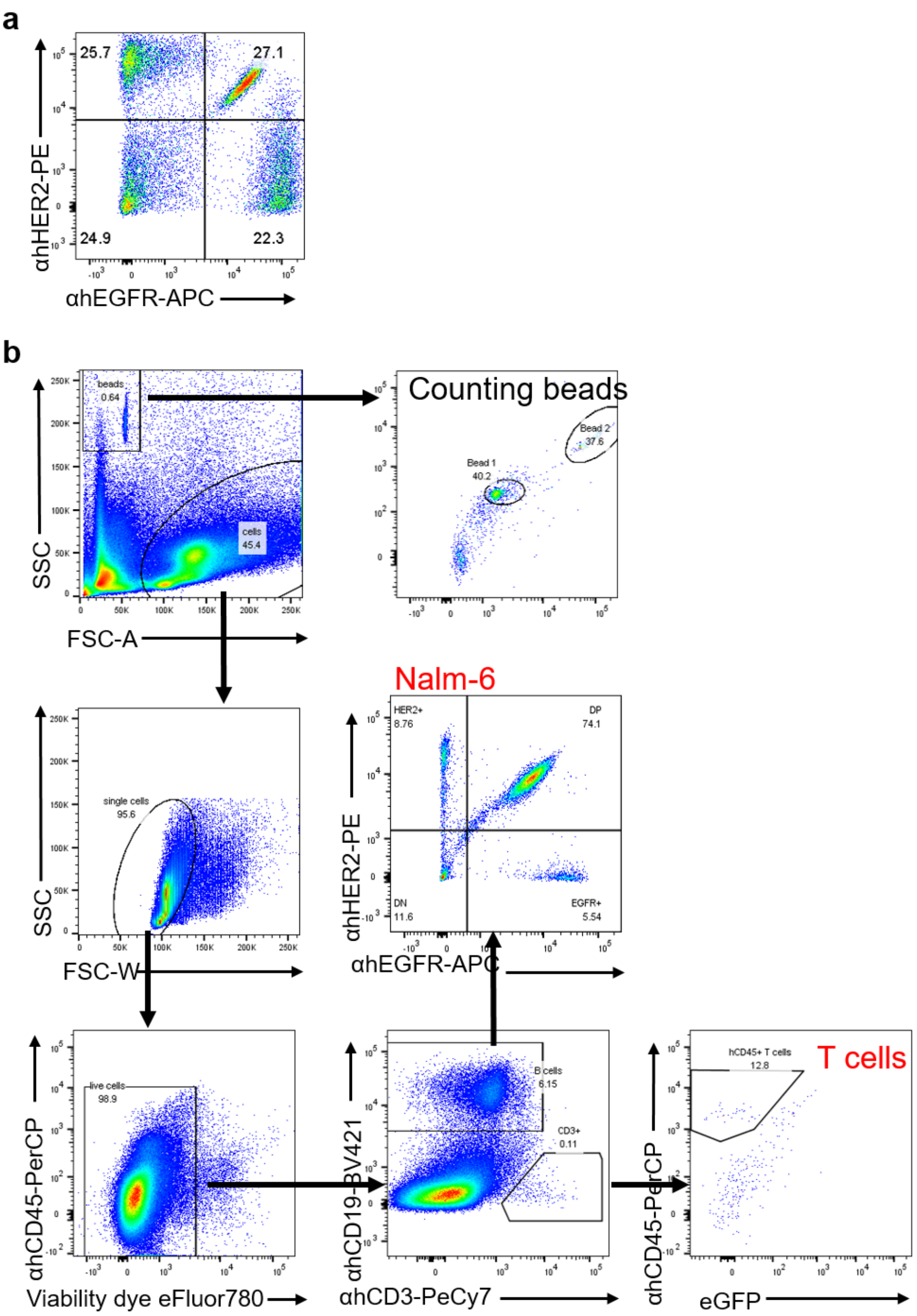

Supplementary Figure 9: Flow cytometric analyses performed during the *in vivo* experiment with the AND-gate AvidCAR shown in Figs. 6c and 6d. (a) Flow cytometric analysis of the 1:1:1:1-mix of Nalm-6, hEGFRt-Nalm-6, hHER2t-Nalm-6 and hHER2t-hEGFRt-Nalm-6 cells, which was administered to the NSG mice. **(b)** Gating strategy in the flow cytometric quantification of Nalm-6 cells and CAR-T cells isolated from organs of NSG mice on day 13 after tumor cell injection. Shown is the bone marrow of one representative mouse.

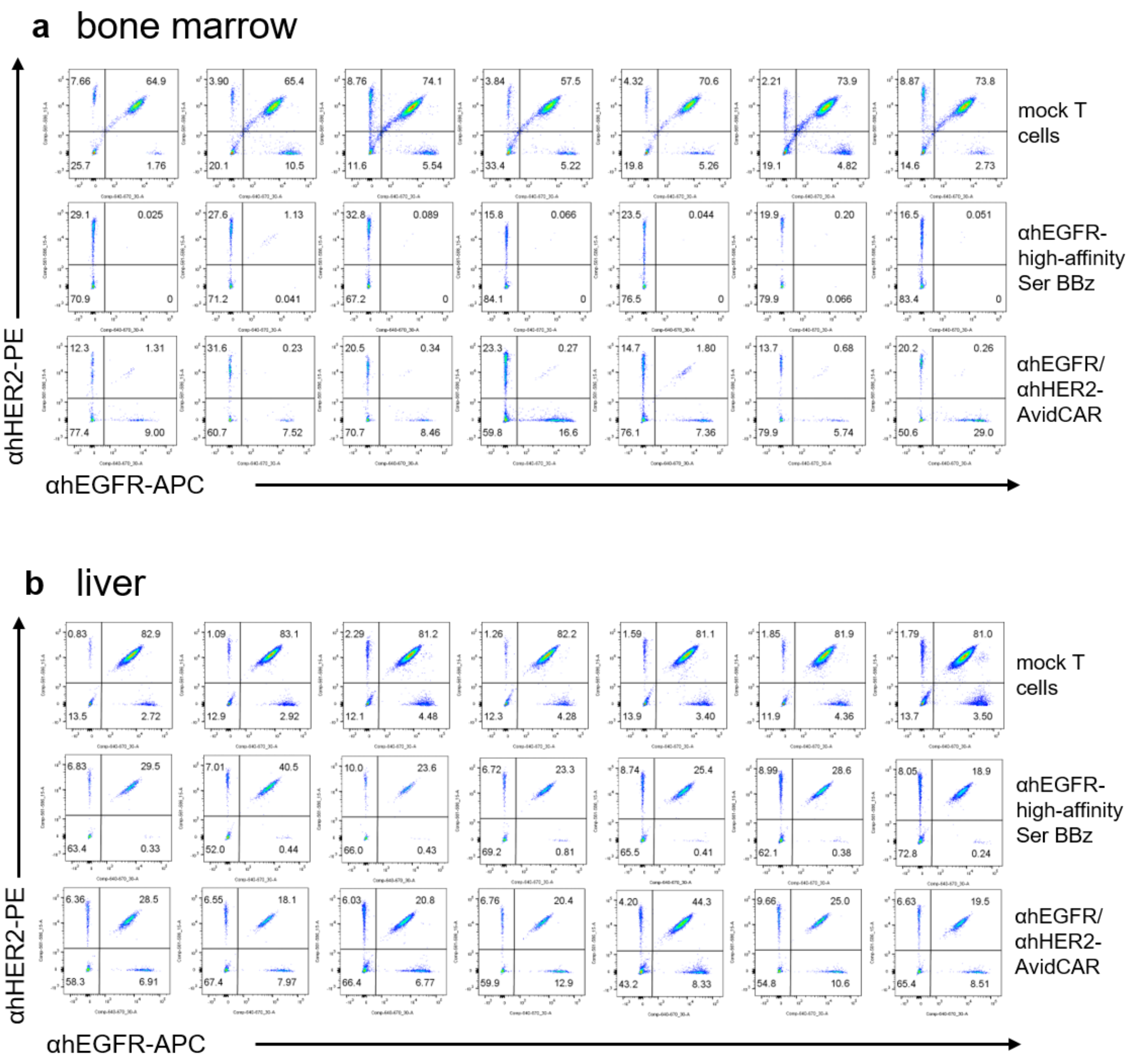

Supplementary Figure 10: Flow cytometric analyses performed during the *in vivo* experiment with the AND-gate AvidCAR shown in Figs. 6c and 6d. Quantification of the ratio of Nalm-6, hEGFRt-Nalm-6, hHER2t-Nalm-6 and hHER2t-hEGFRt-Nalm-6 cells in **(a)** the bone marrow or **(b)** liver of individual mice. Each dot plot represents a different animal (n=7 per group). The gating strategy is shown in Supplementary Fig. 9.

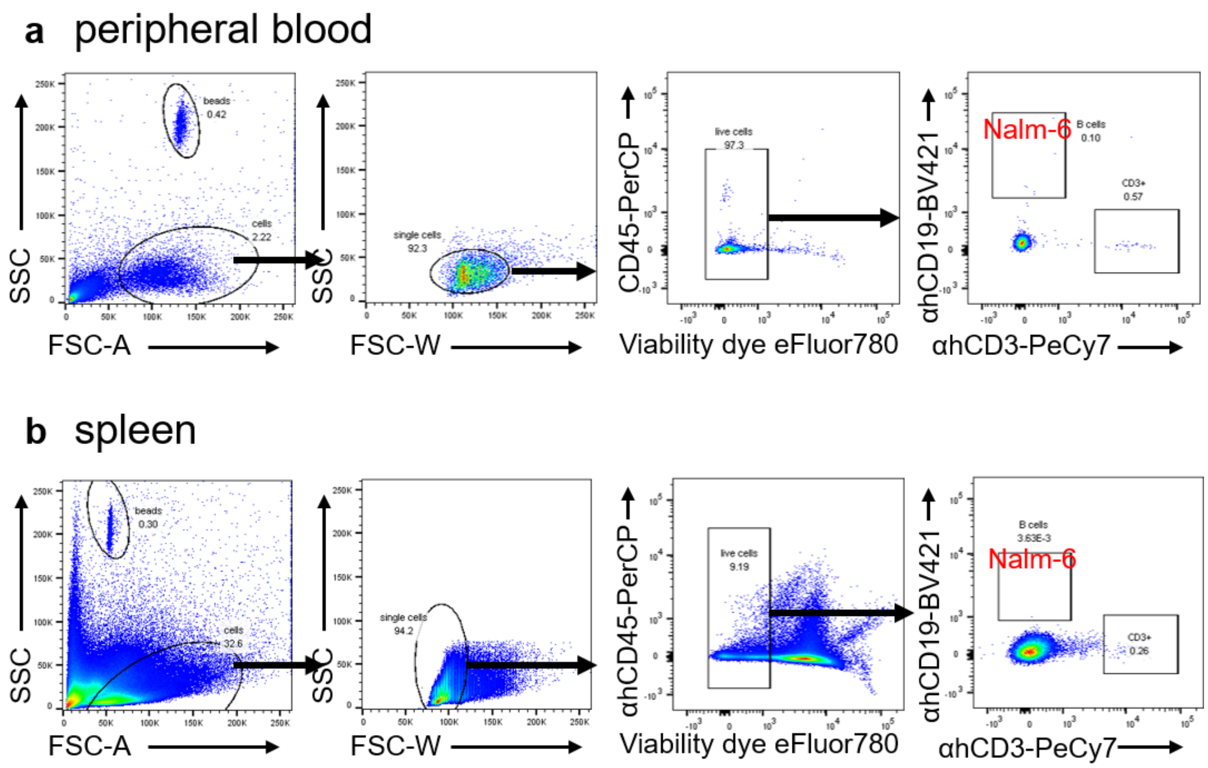

Supplementary Figure 11: Flow cytometric analyses performed during the *in vivo* experiment with the AND-gate AvidCAR shown in Figs. 6c and 6d. Quantification of the different Nalm-6 cells and CAR-T cells isolated from peripheral blood **(a)** and spleen **(b)** of NSG mice on day 13 after tumor cell injection. A representative mouse is shown in each case.

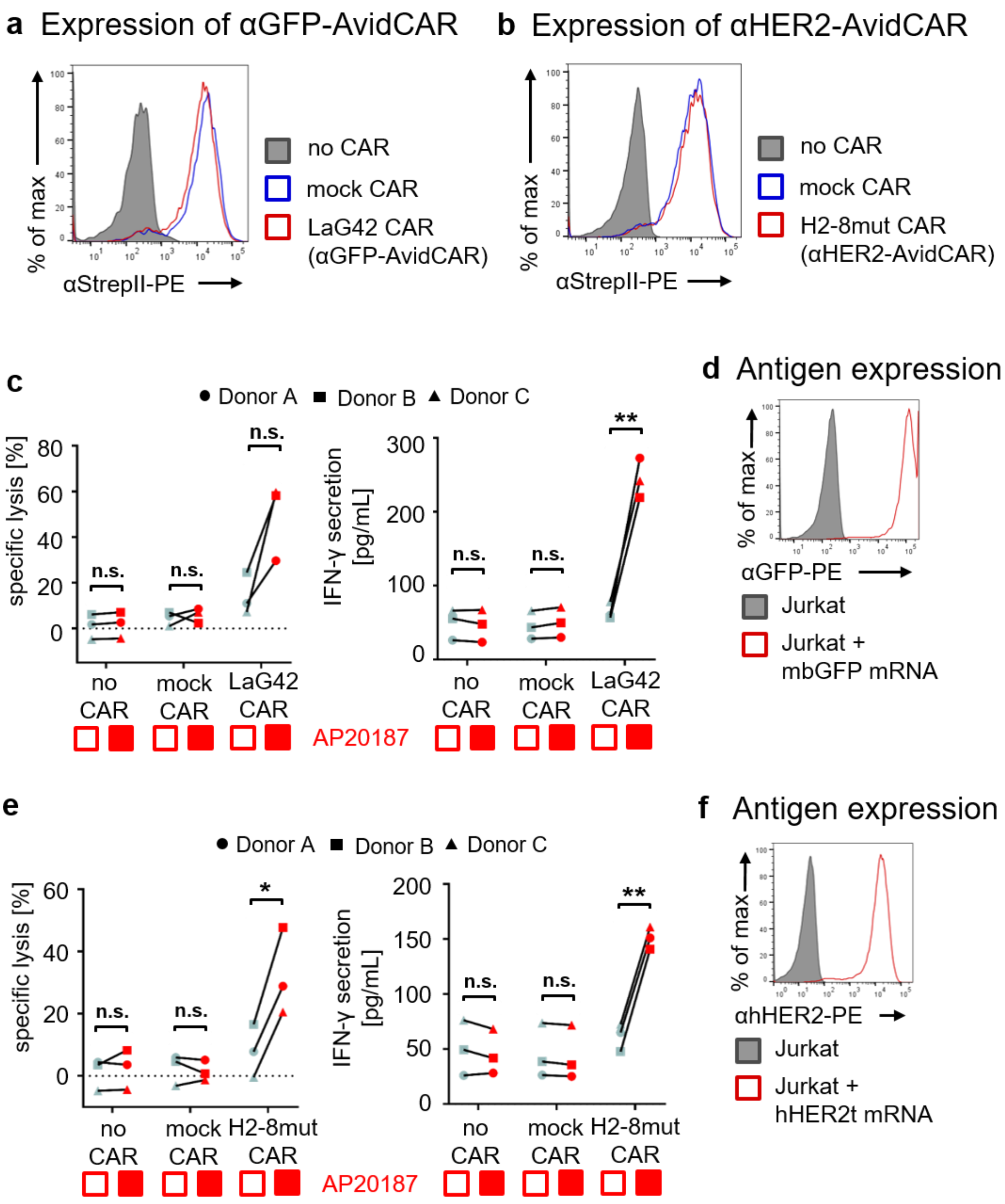

Supplementary Figure 12: AvidCARs containing nanobody-based antigen binding domains. Expression of nanobody-based ON-switch AvidCARs with specificity for **(a)** membrane-bound GFP (mbGFP) (LaG42 CAR; SEQ-ID 27) or **(b)** hHER2 (H2-8mut CAR; SEQ-ID 28) in primary human T cells. An irrelevant nanobody-based low-affinity CAR (mock CAR) served as control. All CARs contained a FKBP12-F36V domain for conditional homodimerization. **(c-f)** Function of nanobody-based ON-switch AvidCARs. Electroporated T cells were co-cultured with Jurkat cells expressing the respective target antigen (E:T ratio 2:1) and lysis of the target cells (Luciferase-based assay) and IFN-γ production was

determined. Expanded T cells were either mock electroporated (no CAR) or electroporated with mRNA of and an irrelevant CAR (mock CAR) or an ON-switch AvidCAR as indicated. Two experiments were performed with 3 different donors in each case [* = $p<0.05$, ** = $p<0.01$, calculated by two-tailed paired *t* test (target cell lysis) or two-tailed ratio-paired *t* test (IFN-γ production)]. **(d and f)** Expression of the target antigens mbGFP (SEQ-ID 29) and hHER2t (SEQ-ID 22) in Jurkat cells. Exact p values are given in Supplementary Data 2. Source data are provided as a Source Data file.
