# Supplementary material for: Engineering AvidCARs for combinatorial antigen recognition and reversible control of CAR function
Source: Nat Commun. 2020 Aug 20;11:4166. doi: 10.1038/s41467-020-17970-3 (PMC7441178; doi:10.1038/s41467-020-17970-3)
Supplement: Supplementary file 7 — Reporting Summary [file 41467_2020_17970_MOESM7_ESM.pdf]

nature research

Corresponding author(s): Michael W. Traxlmayr and Manfred Lehner

Last updated by author(s): Jul 10, 2020

Reporting Summary

Nature Research wishes to improve the reproducibility of the work that we publish. This form provides structure for consistency and transparency in reporting. For further information on Nature Research policies, see our Editorial Policies and the Editorial Policy Checklist.

Statistics

For all statistical analyses, confirm that the following items are present in the figure legend, table legend, main text, or Methods section.

n/a	Confirmed	
☐	☒	The exact sample size (*n*) for each experimental group/condition, given as a discrete number and unit of measurement
☐	☒	A statement on whether measurements were taken from distinct samples or whether the same sample was measured repeatedly
☐	☒	The statistical test(s) used AND whether they are one- or two-sided<br>*Only common tests should be described solely by name; describe more complex techniques in the Methods section.*
☒	☐	A description of all covariates tested
☒	☐	A description of any assumptions or corrections, such as tests of normality and adjustment for multiple comparisons
☐	☒	A full description of the statistical parameters including central tendency (e.g. means) or other basic estimates (e.g. regression coefficient) AND variation (e.g. standard deviation) or associated estimates of uncertainty (e.g. confidence intervals)
☐	☒	For null hypothesis testing, the test statistic (e.g. *F*, *t*, *r*) with confidence intervals, effect sizes, degrees of freedom and *P* value noted<br>*Give P values as exact values whenever suitable.*
☒	☐	For Bayesian analysis, information on the choice of priors and Markov chain Monte Carlo settings
☒	☐	For hierarchical and complex designs, identification of the appropriate level for tests and full reporting of outcomes
☒	☐	Estimates of effect sizes (e.g. Cohen's *d*, Pearson's *r*), indicating how they were calculated

Our web collection on statistics for biologists contains articles on many of the points above.

Software and code

Policy information about availability of computer code

Data collection	Flow cytometric data was collected using BD FACSDivaTM Software V8.0.1. Bioluminescence data was collected using the Living Image Software V4.5.2 (PerkinElmer). Western Blot data was collected using the Odyssey Infrared Imaging System software V3.0 (LI-COR). SPR data was collected using the Biacore T200 Control Software V2.0.2.
Data analysis	Microsoft Excel for Windows 2010, FlowJo V10.6.1, GraphPad Prism V7, Living Image Software V4.5.2, MatLab R2017b, Biacore T200 Evaluation Software V3.1, SAS 9.4, BioNetGen V2.3.2

For manuscripts utilizing custom algorithms or software that are central to the research but not yet described in published literature, software must be made available to editors and reviewers. We strongly encourage code deposition in a community repository (e.g. GitHub). See the Nature Research guidelines for submitting code & software for further information.

Data

Policy information about availability of data

All manuscripts must include a data availability statement. This statement should provide the following information, where applicable:

- Accession codes, unique identifiers, or web links for publicly available datasets
- A list of figures that have associated raw data
- A description of any restrictions on data availability

The authors declare that all data supporting the findings of this study are available within the article and its Supplementary Information files or from the corresponding authors upon reasonable request. The source data for the Figs. 2a, 2c, 2e, 3b, 3e, 3g, 4b, 4d, 5a-c, 6b and 6d, as well as Supplementary Figs. 2c, 4a-b, 4d, 5e, 6a-b, 12c and 12e are provided as a Source Data file. Source data are provided with this paper.

Field-specific reporting

Please select the one below that is the best fit for your research. If you are not sure, read the appropriate sections before making your selection.

- [x] Life sciences
- [ ] Behavioural & social sciences
- [ ] Ecological, evolutionary & environmental sciences

For a reference copy of the document with all sections, see nature.com/documents/nr-reporting-summary-flat.pdf

Life sciences study design

All studies must disclose on these points even when the disclosure is negative.

Sample size	Sample size calculation was performed using the following variables: alpha = 0.05, power = 90%. Due to low variance in our experimental setup, the power calculation determined that 5 animals per group was sufficient to determine statistical significance.
Data exclusions	No data were excluded from the analysis.
Replication	The replication number is indicated in the legend of corresponding figures where applicable. All attempts at replication were successful.
Randomization	For the in vivo tumor model in Figure 6d, equal mean tumor burden was achieved by the administration of a 1:1:1:1 mixture of Nalm-6, hEGFRt-Nalm-6, hHER2t-Nalm-6 and hHER2t-hEGFRt-Nalm-6 cells. For the in vivo tumor models in Figure 5c, mice were randomized to ensure equal mean tumor burden before T cell transfer. For the in vivo tumor model in Figure 5d, no randomization was performed due to technical limitations.
Blinding	No blinding was performed. Objective assessment of the well-being of mice was achieved by the usage of stringent score sheets and the evaluation by one trained person.

Reporting for specific materials, systems and methods

We require information from authors about some types of materials, experimental systems and methods used in many studies. Here, indicate whether each material, system or method listed is relevant to your study. If you are not sure if a list item applies to your research, read the appropriate section before selecting a response.

Materials & experimental systems

n/a	Involved in the study	
[ ]	[x]	Antibodies
[ ]	[x]	Eukaryotic cell lines
[x]	[ ]	Palaeontology and archaeology
[ ]	[x]	Animals and other organisms
[ ]	[x]	Human research participants
[x]	[ ]	Clinical data
[x]	[ ]	Dual use research of concern

Methods

n/a	Involved in the study	
[x]	[ ]	ChIP-seq
[ ]	[x]	Flow cytometry
[x]	[ ]	MRI-based neuroimaging

Antibodies

Antibodies used	The following conjugated antibodies were used:<br>Flow cytometry<br>anti-human HER2-PE (clone 24D2, BioLegend, #324405, lot no: B215135), anti-human EGFR-PE (clone AY13, BioLegend, #352903, lot no: B208129), anti-human EGFR-APC (clone AY13, BioLegend, #352905, lot no: B208903), anti FLAG-PE (clone L5, BioLegend, #637309, lot no: B229834), anti FLAG-APC (clone L5, BioLegend, #637307, lot no: B259897), anti StrepII-Biotin (clone 5A9F9, GenScript, #A01737, lot no: 11B000265), anti-human IgG Fc-PE (clone JDC-10, Southern Biotech, #9040-09, lot no: K3812-V383Z), anti-human CD19-BV421 (clone HIB19, BioLegend, #302233, lot no: B275424), anti-human CD45-PerCP (clone 2D1; BD Biosciences, #345809, lot no: 9344716), anti-human CD3-PE-C7 (clone SK7; BD Biosciences, #557851, lot no: 7116624), anti GFP-PE (clone FM264G; BioLegend, #338003, lot no: B292202), streptavidin-APC (eBioscience, #17-4317-82, lot no: E07261-1632), streptavidin-PE (eBioscience, #12-4317-87, lot no: 4306317), Biotin-Protein L (GenScript, #JP_M00097)<br>Western Blot<br>anti-human EGFR (clone 528, Thermo Scientific, #MA5-12875, lot no: RL2311818), anti-human GAPDH (clone N/A, Abcam, #ab9485), goat anti-mouse IgG (H+L) Secondary Antibody, HRP conjugate (clone N/A, #62-6520, lot no: TG273230), goat anti-rabbit IgG (H+L) Secondary Antibody, DyLight 800 (clone N/A, Thermo Scientific, #SA5-35571)
Validation	All the antibodies are validated for use in flow cytometry or western blotting, respectively. In our laboratory, antibody-specific staining was compared to isotype and no staining control samples.<br>anti-human HER2-PE (clone 24D2) was validated here https://www.biolegend.com/de-at/products/pe-anti-human-cd340-erbb2-her-2-antibody-3766<br>anti-human EGFR-PE (clone AY13) was validated here https://www.biolegend.com/de-at/products/pe-anti-human-egfr-

antibody-7432
anti-human EGFR-APC (clone AY13) was validated here https://www.biolegend.com/de-at/search-results/apc-anti-human-egfr-antibody-7714
anti FLAG-PE (clone L5) was validated here https://www.biolegend.com/de-at/products/pe-anti-dykddddk-tag-antibody-9383
anti FLAG-APC (clone L5) was validated here https://www.biolegend.com/de-at/search-results/apc-anti-dykddddk-tag-antibody-8099
anti StrepII-Biotin (clone 5A9F9) was validated here https://www.genscript.com/antibody/A01737-THE_sup_TM_sup_NWSHPQFEK_Tag_Antibody_Biotin_mAb_Mouse.html
anti-human IgG Fc-PE (clone JDC-10) was validated here https://www.southernbiotech.com/?catno=9040-09&type=Monoclonal#&panel2-1
anti-human CD19-BV421 (clone HIB19) was validated here https://www.biolegend.com/de-at/products/brilliant-violet-421-anti-human-cd19-antibody-7144
anti-human CD45-PerCP (clone 2D1) was validated here https://www.bdbiosciences.com/us/applications/research/stem-cell-research/cancer-research/human/percp-mouse-anti-human-cd45-2d1/p/347464
anti-human CD3-PE-C7 (clone SK7) was validated here https://www.bdbiosciences.com/us/applications/research/t-cell-immunology/th-1-cells/surface-markers/human/pe-cy7-mouse-anti-human-cd3-sk7-also-known-as-leu-4/p/557851
anti GFP-PE (clone FM264G) was validated here https://www.biolegend.com/de-at/products/pe-anti-gfp-antibody-5479
streptavidin-PE was validated here https://www.thermofisher.com/order/catalog/product/12-4317-87?SID=srch-hj-12-4317-87#/12-4317-87?SID=srch-hj-12-4317-87
streptavidin-APC was validated here https://www.thermofisher.com/order/catalog/product/17-4317-82?SID=srch-hj-17-4317-82#/17-4317-82?SID=srch-hj-17-4317-82
Biotin-Protein L was validated here https://www.genscript.com/molecule/M00097-Biotin_Protein_L.html
anti-human EGFR (clone 528) was validated here https://www.thermofisher.com/antibody/product/EGFR-Antibody-clone-528-Monoclonal/MA5-12875
anti-human GAPDH was validated here https://www.abcam.com/gapdh-antibody-loading-control-ab9485.html
goat anti-mouse IgG (H+L) Secondary Antibody, HRP conjugate was validated here https://www.thermofisher.com/antibody/product/Goat-anti-Mouse-IgG-H-L-Secondary-Antibody-Polyclonal/62-6520
goat anti-rabbit IgG (H+L) Secondary Antibody, DyLight 800 was validated here https://www.thermofisher.com/antibody/product/Goat-anti-Rabbit-IgG-H-L-Secondary-Antibody-Polyclonal/SA5-35571

Eukaryotic cell lines

Policy information about cell lines

Cell line source(s)	Jurkat and Nalm-6 cell lines were purchased from DSMZ. Lenti-X 293T cells were purchased from Takara.
Authentication	Jurkat and Nalm-6 cell lines were authenticated by Single Nucleotide Polymorphism (SNP)-profiling (Multiplexion, Germany). Lenti-X 293T cells (Takara) were not authenticated.
Mycoplasma contamination	All cell lines tested negative for mycoplasma.
Commonly misidentified lines (See ICLAC register)	No commonly misidentified cell line was used.

Animals and other organisms

Policy information about studies involving animals; ARRIVE guidelines recommended for reporting animal research

Laboratory animals	6-20 week old male and female NOD.Cg-Prkdcscid Il2rgtm1WJl/SzJ (NSG) mice (The Jackson Laboratory) were used where indicated.
Wild animals	This study did not involve wild animals.
Field-collected samples	This study did not involve samples collected in the field.
Ethics oversight	All procedures were approved by the Magistratsabteilung 58, Vienna, Austria (GZ: 319093 /2014/16) and the Federal Ministry Republic of Austria for Education, Science and Research (BMBWF-66.009/0243-V/3b/2019).

Note that full information on the approval of the study protocol must also be provided in the manuscript.

Human research participants

Policy information about studies involving human research participants

Population characteristics	Buffy coats from anonymous healthy donors were purchased from the Red Cross, Vienna. The researchers were blind to any covariate characteristics.
Recruitment	There was no recruitment of participants. Buffy coats were purchased from the Red Cross, Vienna, as described.
Ethics oversight	N/A

Note that full information on the approval of the study protocol must also be provided in the manuscript.

Flow Cytometry

Plots

Confirm that:

- [x] The axis labels state the marker and fluorochrome used (e.g. CD4-FITC).
- [x] The axis scales are clearly visible. Include numbers along axes only for bottom left plot of group (a 'group' is an analysis of identical markers).
- [x] All plots are contour plots with outliers or pseudocolor plots.
- [x] A numerical value for number of cells or percentage (with statistics) is provided.

Methodology

Sample preparation	Buffy coats from de-identified healthy donors were isolated and purified as described in Methods. Sample preparation was performed as described in Methods.
Instrument	BD LSRFortessaTM
Software	Data was collected using BD FACSDivaTM Software and processed using FlowJo V10.6.1 (FlowJo, Inc.).
Cell population abundance	No sorting was performed.
Gating strategy	Nonviable cells were excluded based upon FSC/SSC gating or a viability dye.

- [x] Tick this box to confirm that a figure exemplifying the gating strategy is provided in the Supplementary Information.
