# Supplementary material for: Engineering AvidCARs for combinatorial antigen recognition and reversible control of CAR function
Source: Nat Commun. 2020 Aug 20;11:4166. doi: 10.1038/s41467-020-17970-3 (PMC7441178; doi:10.1038/s41467-020-17970-3)
Supplement: Supplementary file 8 — Description of Additional Supplementary Files [file 41467_2020_17970_MOESM8_ESM.pdf]

Title: Supplementary Data 1
Description: This table contains a detailed list of constructs used in this study and the respective sequence information.

Title: Supplementary Data 2
Description: This table contains the exact p values reported in this study.

Title: Supplementary Data 3
Description: This table contains the DNA sequences of oligonucleotides used in this study. Sequences are presented from the 5' end to the 3' end.

Title: Supplementary Data 4
Description: This folder contains the bngl file defining the mathematical model shown in Supplementary Fig. 3 along with the m-file used to solve it.
